# Do 20-minute neighbourhoods moderate associations between work and commute hours with food consumption?

**DOI:** 10.1017/S1368980023000587

**Published:** 2023-10

**Authors:** Laura Helena Oostenbach, Karen Elaine Lamb, David Crawford, Anna Timperio, Lukar Ezra Thornton

**Affiliations:** 1 Institute for Physical Activity and Nutrition, School of Exercise and Nutrition Sciences, Deakin University, 1 Gheringhap Street, Geelong 3220, Australia; 2 Melbourne School of Population and Global Health, University of Melbourne, Carlton, Melbourne, Australia; 3 Department of Marketing, Faculty of Business and Economics, University of Antwerp, Antwerp, Belgium

**Keywords:** Food consumption, Work hours, Commute time, 20-minute neighbourhood, Australia

## Abstract

**Objective:**

To examine associations between work and commute hours with food consumption and test whether neighbourhood type (20-minute neighbourhood (20MN)/non-20MN) moderate associations.

**Design:**

Cross-sectional analysis of the Places and Locations for Activity and Nutrition study (ProjectPLAN). Exposures were work hours (not working (0 h), working up to full-time (1–38 h/week), working overtime (> 38 h/week)), and among those employed, combined weekly work and commute hours (continuous). Outcomes were usual consumption of fruit, vegetables, takeaway food, snacks and soft drinks, and number of discretionary food types (takeaway, snacks and soft drinks) consumed weekly. Generalised linear models were fitted to examine associations between each exposure and outcome. The moderating role of neighbourhood type was examined through interaction terms between each exposure and neighbourhood type (20MN/non-20MN).

**Setting:**

Melbourne and Adelaide, Australia, 2018–2019.

**Participants:**

Adults ≥ 18 years old (*n* 769).

**Results:**

Although all confidence intervals contained the null, overall, patterns suggested non-workers and overtime workers have less healthy food behaviours than up-to-full-time workers. Among those employed, analysis of continuous work and commute hours data suggested longer work and commute hours were positively associated with takeaway consumption (OR = 1·014, 95 % CI 0·999, 1·030, *P*-value = 0·066). Patterns of better behaviours were observed across most outcomes for those in 20MN than non-20MN. However, differences in associations between work and commute hours with food consumption across neighbourhood type were negligible.

**Conclusions:**

Longer work and commute hours may induce poorer food behaviours. There was weak evidence to suggest 20MN moderate associations between work and commute hours with food consumption, although behaviours appeared healthier for those in 20MN.

In Australia, the labour force participation rate was 67 % for women and 78 % for men aged 20–74 years old in 2017–18^(1)^. Work-related time demands, including work hours and commuting to and from work, can place demands on working individuals’ time, posing a risk of time scarcity^(2)^. Almost half of Australians who work full-time always or often feel rushed or pressed for time, compared to 40 % of those employed part time, 30 % of those unemployed and 17 % of those not in the labour force^(3)^, and balancing work with family responsibilities is the most common main reason for feeling rushed or pressured for time^(4)^. Time scarcity (i.e. lacking enough time to undertake everyday activities) can limit engagement in health-related activities^(2)^ and healthy food practices^(5)^, including selecting, purchasing, preparing and eating healthy foods^(6)^. Previous cross-sectional studies have also linked time scarcity to unhealthy food consumption, including higher consumption of convenience food such as ultra-processed dinners, snacks, soft drinks and fast food^(7)^ and lower consumption of fruits and vegetables^(8)^. Research has shown that work hours are associated with time-related barriers to healthful eating among adults^(9)^ and their children^(10)^. However, overall, findings on the specific role of employment-related time demands in food practices are mixed^(9,11–15)^ and mostly based on studies conducted in the United States (US)^(9,11–13)^.

Workers with limited available time (owing to their work hours and commute times) may interact with their residential neighbourhood differently to those who have more time available. It is plausible that those who are time scarce favour quick and convenient options such as drive-through takeaway outlets or purchasing fast foods instead of buying fresh foods, which requires time invested into visiting the retailer and selecting and preparing the food. However, studies have so far not incorporated the potential role of neighbourhood design when considering links between work hours and commute time with food consumption.

To adapt to urban growth and ensure neighbourhood liveability, cities worldwide have developed plans to promote compact city designs, encouraging localised lifestyles such as accessing healthy foods locally, potentially benefiting population health^(16,17)^. For example, in Australia, the cities of Melbourne and Adelaide have included the creation of compact and walkable neighbourhoods in urban development plans^(18,19)^. While both cities focus on localised and healthy living, Melbourne has adopted a concept called ‘20-minute neighbourhoods’ (20MN)^(16,19)^. This concept aims to support everyday non-work-related needs within a short distance from home via access to co-located amenities and services^(16)^. While health benefits such as healthier diets^(20)^ have been projected, it remains unknown whether 20MN benefit working individuals with long work hours and commute times who may have limited time to interact with their local neighbourhood.

This study examines the role of work hours and commute time in food consumption, and whether these associations are moderated by 20MN. We expect those working overtime to have less healthy food behaviours than those working shorter hours. We also expect less healthful food behaviours among those working as their combined work and commute hours increase. However, we hypothesise that detrimental associations are attenuated for those living in a 20MN, as these neighbourhoods may facilitate easier access to healthy foods^(16)^. This study will contribute to our understanding of food practices in the working population, informing policies linked to workers’ health and employment arrangements, such as flexible work hours and telecommuting. It will also provide evidence as to whether 20MN alleviate negative impacts of employment-related time demands on food practices, informing urban planning decisions around neighbourhood design.

## Methods

### Sample

Participants (*n* 769 adults ≥ 18 years old) from the 2018–2019 cross-sectional Places and Locations for Activity and Nutrition study (ProjectPLAN) who completed the ‘food survey’ were examined. ProjectPLAN aimed to assess relationships between having a 20MN with physical activity and food practices. Participants were randomly selected through stratified random sampling by city (Melbourne/Adelaide), neighbourhood type (20MN/non-20MN) and neighbourhood socio-economic status (SES) (high/low). The 2016 Australian Bureau of Statistics (ABS) Greater Capital City Statistical Areas were used to determine the spatial extent of each city^(21)^. The detailed operationalisation of a 20MN is described elsewhere^(22)^. Briefly, five domains were identified, drawing on Plan Melbourne’s definition of a 20MN^(19)^: (1) healthy food; (2) community facilities; (3) recreation facilities; (4) public open space and (5) public transport. These five domains were determined from eleven individual attributes. For example, the healthy food domain required access to a large supermarket OR a small supermarket and greengrocer within a 1·5 km pedestrian network distance. 20MN were defined as areas with access to each of the five domains, that is, with high levels of service and amenity provision^(22)^. Non-20MN were areas with low levels of services and amenities (≤ 5 individual attributes, i.e., low levels of service and amenity provision). Neighbourhood SES was based on the 2016 ABS Socio-Economic Indexes for Areas (SEIFA) Index of Relative Socio-economic Advantage and Disadvantage (IRSAD)^(23)^. The IRSAD summarises information on economic and social conditions of individuals and households within an area, including relative advantage and disadvantage measures, using income and occupation data from the Australian census^(24)^. Low SES areas were based on the Statistical Areas level 1 (SA1) SEIFA IRSAD decile 1, 2 or 3 that had to be within larger statistical areas (SA2) of decile 1, 2 or 3. SA1 within SA2 boundaries were extracted to represent small areas of low SES within larger areas that also had low SES. High SES was classified as SA1 with a SEIFA IRSAD decile of 8, 9 or 10 within an SA2 of decile 8, 9 or 10^(24)^. Address points within residential Mesh Blocks (i.e. the smallest geographic areas defined by the ABS) were intersected with the neighbourhood type layer and neighbourhood SES layer, and a random sample of address points (sourced from routinely available government data sources^(25,26)^) was selected from each combination of city (Melbourne/Adelaide), neighbourhood type (20MN/non-20MN) and neighbourhood SES (high/low).

A mass mail-out of >10 000 letters was undertaken for the food survey. Households at selected address points received a mailed invitation to participate in either a physical activity or food survey which contained a URL and unique code to access the Plain Language Statement, consent form and survey. Additional mail outs to eligible addresses were conducted to maximise recruitment in strata with lower responses (e.g. low SES neighbourhoods). Eligible food survey participants were ≥ 18 years old, still living at the same address to which the invitation mailed and mainly or jointly responsible for the household food shopping.

### Outcomes

Outcomes included usual (1) fruit consumption (serves/day), (2) vegetables consumption (serves/day), (3) hot takeaway food consumption (e.g. fish and chips, burgers, pizza, sausage rolls, meat pies, fried chicken) (< once/week, once/week, > once/week), (4) snack consumption (e.g. chocolate, lollies/sweets/candy, cake, chips, ice cream, donuts, sweet biscuits) (< once/week, 1–2 times/week, 3–4 times/week, ≥ 5 times/week) and (5) soft drink consumption (sugar-sweetened beverages) (< once/week, 1–2 times/week, ≥ 3 times/week). Items were constructed using previously published and validated surveys^(27,28)^. Additionally, cumulative unhealthy food practices were explored through the number of discretionary food types (hot takeaway, snacks and soft drinks) consumed at least weekly. Daily serves of fruit and vegetables were treated as count data ranging from 0 to 8. Similarly, the number of discretionary food types consumed weekly was treated as count data ranging from 0 to 3. Takeaway, snack and soft drink consumption were treated as ordered categorical variables. Survey questions and operationalisation are detailed in Additional file 1.

### Exposures

Participants reported whether they were employed (including self-employed) in a paid job or unemployed in a usual week. Unemployed included those looking for work, homemakers, students or retirees. Those who reported being employed were asked their number of work hours in all their paid jobs in a normal week. Employed participants who reported usually travelling to the same work location or to many different work locations were further asked about their usual commute time. The first exposure in this study was usual work hours (not working: 0 h; working up to full-time: 1–38 h/week; working overtime: > 38 h/week). Cut-off points were guided by Fair Work Australia’s definition of full-time and overtime hours^(29)^. Similar cut-off points have been used in previous research looking at links between work hours and health^(30)^. The second exposure was combined usual weekly work and commute hours (continuous) only among those in the workforce (see Additional file 1 for survey questions and full operationalisation).

### Confounders

Potential confounders were age (years), gender (male, female, transgender), presence of children in the household (no children, any child ≤ 4 years, only children aged 5–17 years), relationship/living status (single, in a relationship: not living with partner, in a relationship: living with partner), neighbourhood SES (low, high) and city (Melbourne, Adelaide) (Additional file 1).

### Moderator

Neighbourhood type (20MN or non-20MN) was considered as a moderator.

### Statistical analysis

Generalised linear models were fitted to examine associations between each exposure and outcome, with Poisson regression used for each count outcome (daily serves of fruits and vegetables, number of discretionary food types) and ordinal regression for ordinal outcomes (frequency of hot takeaway, snack and soft drink consumption). The proportional odds assumption was assessed using likelihood ratio tests. Combined work and commute hours exposure was examined only among those employed. All models were adjusted for potential confounders by including them as model covariates. The moderating role of 20MN was examined by considering interactions between each exposure and neighbourhood type. Evidence of association between two variables is not a prerequisite to testing for moderation by a third variable. Association between exposure and outcome may sometimes only be elucidated when considered in the context of a third moderating variable^(31)^.

A complete case analysis was performed assuming data were missing completely at random. Sample characteristics for the full sample, complete case and omitted participants are detailed in Additional file 2. Characteristics for the complete case sample appeared to be representative of the full sample.

All analyses were conducted using Stata 16.0.

## Results

### Descriptive characteristics

Of the 769 participants who completed the food survey, 699 (91 %) were included in the complete case analysis. Sixty-one percentage (*n* 427) of the sample were female. The median age of the sample was 57 years old, which also translated to fewer participants with children in their household. Only 14 % (*n* 97) of participants had a child ≤ 4 years living in their household and an additional 13 % (*n* 88) had children aged 5–17 years. Descriptive statistics of all variables included in the analysis are presented in Table 1.

**Table 1:** Descriptive characteristics of participants

	Whole sample (*n* 699)		Employed sub-sample (*n* 378)	
	*n*	%	*n*	%
Fruit consumption (serves/d)				
Median	1·00		2·00	
IQR	1·00, 2·00		1·00, 2·00	
Vegetable consumption (serves/day)				
Median	2·00		2·00	
IQR	1·00, 3·00		2·00, 3·00	
Count of discretionary food types (takeaway, snacks and soft drinks) consumed weekly (*n*)				
0	137	19·6	67	17·7
1	285	40·8	144	38·1
2	166	23·7	94	24·9
3	111	15·9	73	19·3
Takeaway consumption (occasions)				
<1/week	470	67·2	224	59·3
1/week	139	19·9	91	24·1
>1/week	90	12·9	63	16·7
Snack consumption (occasions)				
<1/week	189	27·0	97	25·7
1–2/week	204	29·2	119	31·5
3–4/week	131	18·7	73	19·3
≥5/week	175	25·0	89	23·5
Soft drink consumption (occasions)				
<1/week	488	69·8	262	69·3
1–2/week	107	15·3	62	16·4
≥3/week	104	14·9	54	14·3
Work hours				
Not working (0 h)	321	45·9	N/A	
Working up to full time (1–38 h)	237	33·9	237	62·7
Working overtime (>38 h)	141	20·2	141	37·3
Weekly work and commute hours (employed only)				
Median	N/A		40·46	
IQR			27·50, 47·50	
Age				
Median	57·00		47·00	
IQR	41·00, 67·00		36·00, 57·00	
Gender				
Male	270	38·6	140	37·0
Female	427	61·1	238	63·0
Transgender	2	0·3	0	0·0
Children in household				
No children	514	73·5	248	65·6
Any child ≤ 4 years	97	13·9	61	16·1
Only children aged 5–17 years	88	12·6	69	18·3
Relationship status				
Single	210	30·0	114	30·2
In a relationship: not living with partner	43	6·2	24	6·3
In a relationship: living with partner	446	63·8	240	63·5
Neighbourhood SES				
Low SES	307	43·9	159	42·1
High SES	392	56·1	219	57·9
City				
Melbourne	320	45·8	189	50·0
Adelaide	379	54·2	189	50·0
Neighbourhood design				
20MN	349	49·9	199	52·6
Non-20MN	350	50·1	179	47·4

IQR, inter-quartile range; SES, socio-economic status.

### Associations between work hours and food consumption

**Fig. 1:**
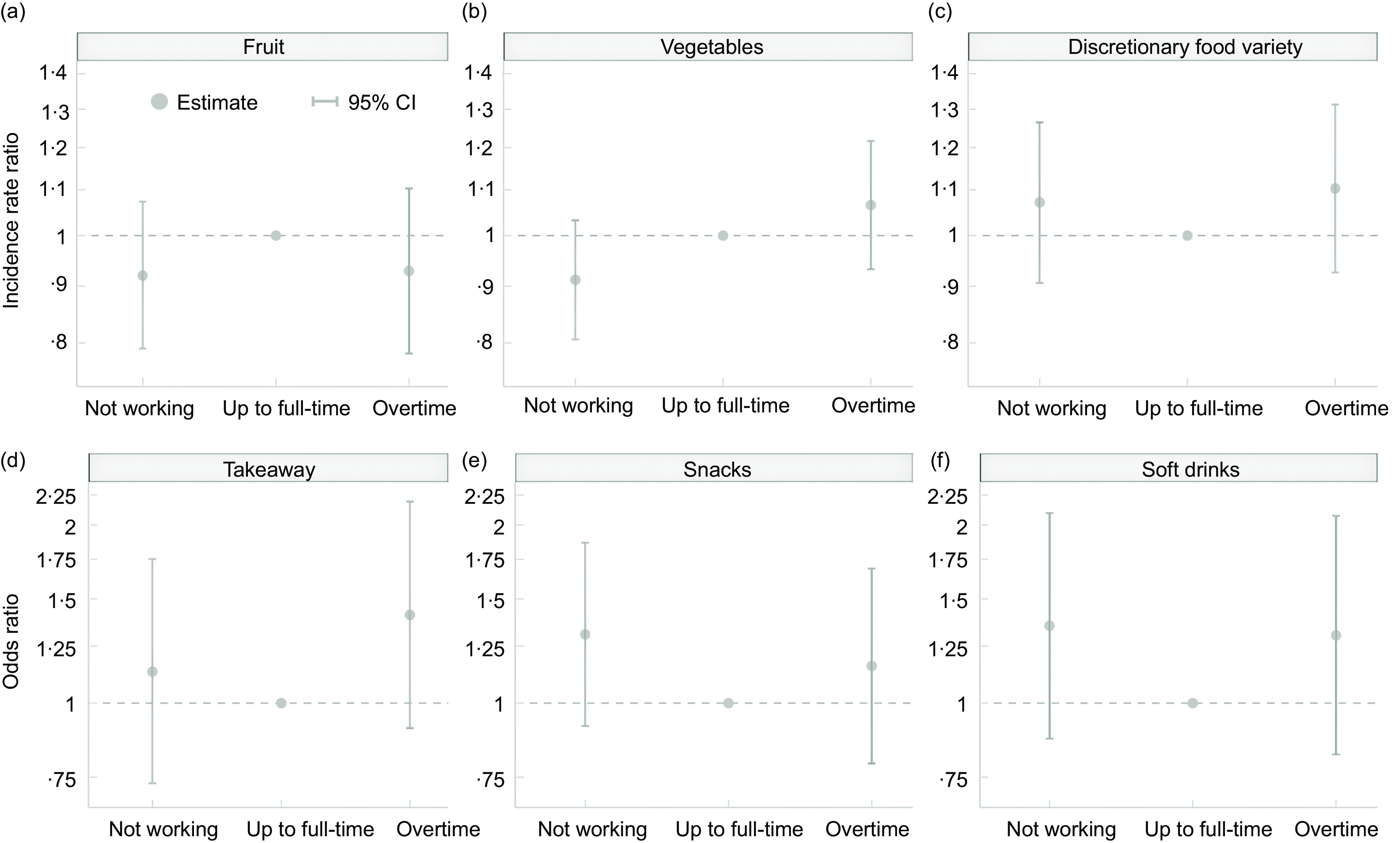
IRR and OR of food consumption per work hours categories (*n* 699). Adjusted Poisson models of daily fruit and vegetables consumption and the variety of discretionary food types consumed weekly (based on the count of discretionary food types (takeaway, snacks and soft drinks) consumed at least weekly) per work hours categories. Adjusted ordinal models of the frequency of takeaway, snack and soft drink consumption per work hours categories. All models adjusted for age, gender, children in household, relationship status, neighbourhood SES, neighbourhood type and city. IRR and OR are displayed on log scale. (Reference category: up to full-time) (not working: 0 h, up to full-time: 1–38 h/week, overtime: >38 h/week). IRR, incidence rate ratio; SES, socio-economic status.

Figure 1 shows the estimated incidence rate ratios (IRR), odds ratios (OR) and confidence intervals (CI) from the adjusted models. While all CI contained the null, patterns were observed across behaviours. Except for vegetable consumption, patterns in the estimated effects suggested both those not working and those working overtime have less healthy dietary behaviours than those working up to full-time, with lower IRR for fruit intake, higher IRR for the variety of discretionary food types consumed weekly and higher odds of takeaway, snacks and soft drink consumption (Fig. 1). Estimates and CI from the adjusted Poisson and ordinal models are also presented in Additional file 3.

### Associations between combined work and commute hours with food consumption

Among those working, although all CI contained the null value, the highest estimated effect of combined work and commute hours was for takeaway consumption, with greater hours associated with higher odds of frequently consuming takeaway foods (OR = 1·014, 95 % CI 0·999, 1·030, *P* = 0·066) (Table 2). Estimates were small, representing the increase in odds for each 1-h increase in work and commute hours, but odds accumulate as time spent working and commuting increases. For example, individuals who work and commute 40 h each week were estimated to have an increased OR of 1·056 for takeaway consumption, that is, a 5·6 % increase in odds.

**Table 2:** IRR and OR of food consumption for combined work and commute hours among employed (*n* 378)

Combined work and commute hours and food consumption			
Poisson regression	IRR	95 % CI	*P*
Daily serves of fruit	0·997	0·992, 1·003	0·306
Daily serves of vegetables	1·001	0·997, 1·006	0·583
Number of different types of discretionary food (i.e. takeaway, snacks and soft drinks) consumed weekly	1·004	0·999, 1·010	0·141
Ordinal regression	OR	95 % CI	*P*
Takeaway consumption	1·014	0·999, 1·030	0·066
Snack consumption	1·009	0·996, 1·022	0·165
Soft drink consumption	1·010	0·994, 1·026	0·231

IRR, incidence rate ratio; SES, socio-economic status. Models adjusted for age, gender, children in household, relationship status, neighbourhood SES, neighbourhood type and city. The estimate represents the increase (or decrease) in IRR or OR per each 1-h increase in combined work and commute hours.

### Moderation by neighbourhood type

**Fig. 2:**
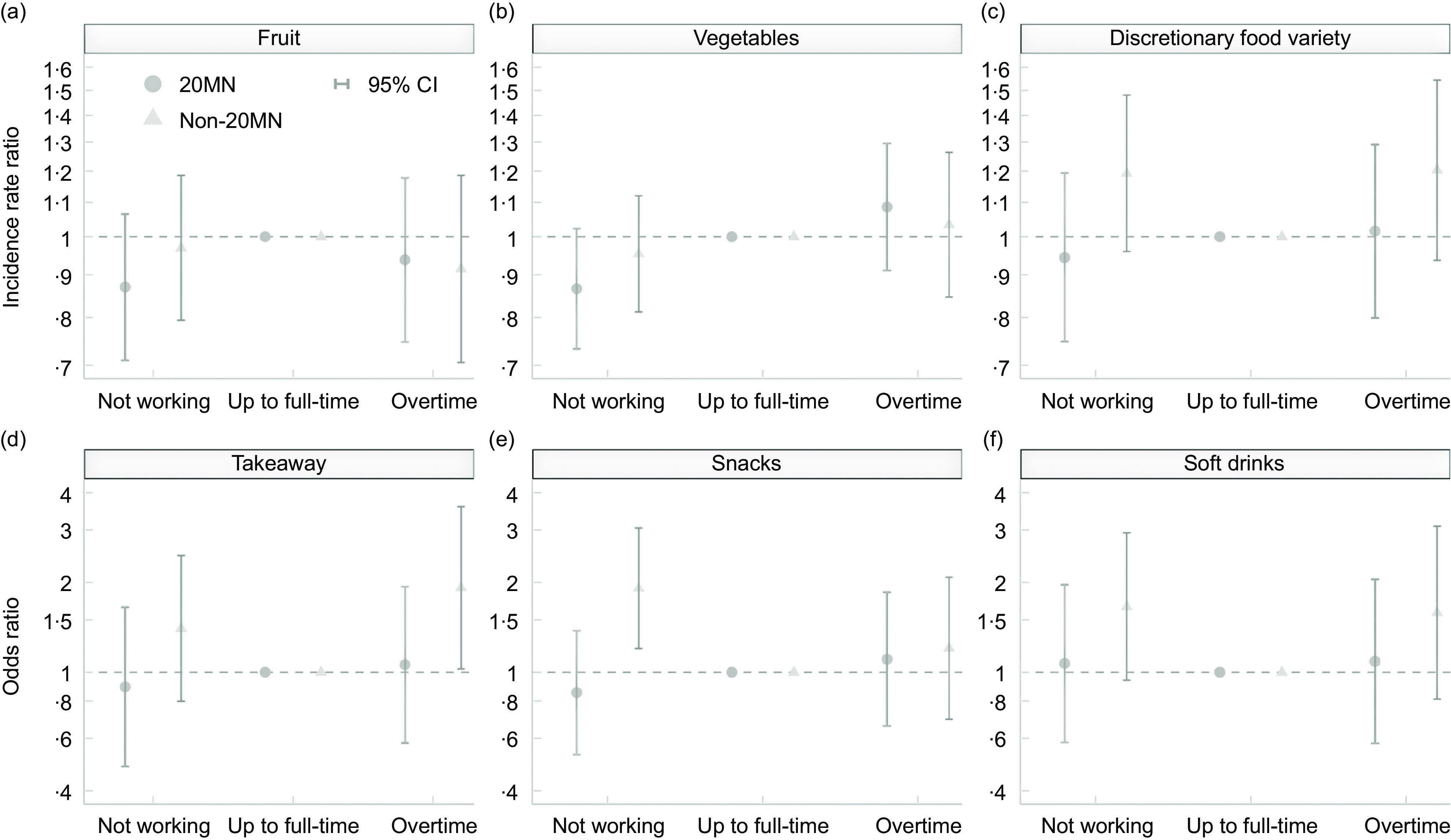
IRR and OR of food consumption by neighbourhood type and work hours categories (*n* 699). Adjusted Poisson models of daily fruit and vegetables consumption and the variety of discretionary food types consumed weekly (based on the count of discretionary food types (takeaway, snacks, and soft drinks) consumed at least weekly) fitted with interaction terms. Adjusted ordinal models of the frequency of takeaway, snack and soft drink consumption fitted with interaction terms. All models adjusted for age, gender, children in household, relationship status, neighbourhood SES and city. IRR and OR are displayed on log scale. (Reference category: up to full-time). (Not working: 0 h, up to full time: 1–38 h/week, overtime: > 38 h/week). 20MN, 20-minute neighbourhood; IRR, incidence rate ratio; SES, socio-economic status.

**Fig. 3:**
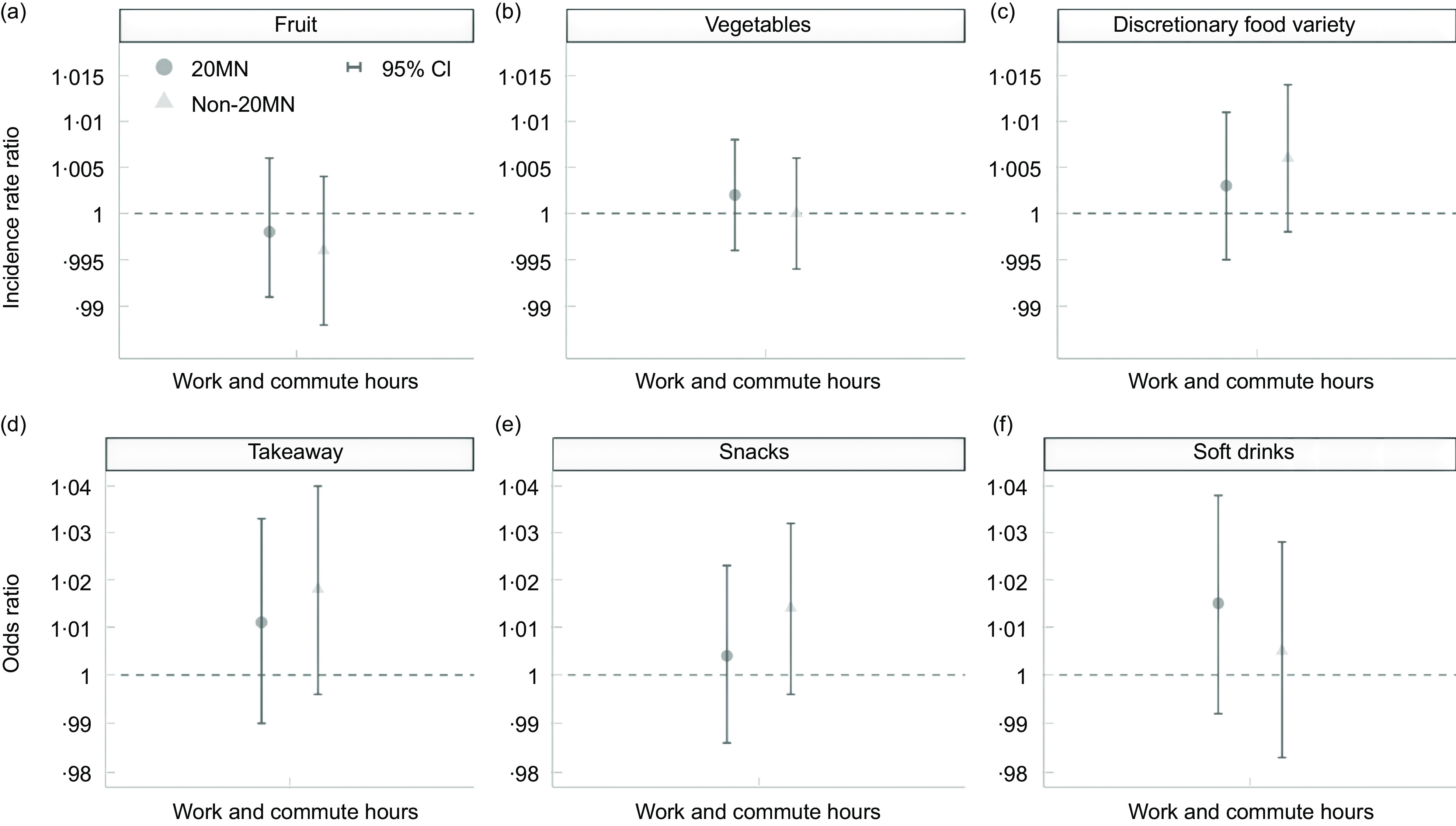
IRR and OR of food consumption by neighbourhood type for combined work and commute hours among those employed (*n* 378). Adjusted Poisson models of daily fruit and vegetables consumption and the variety of discretionary food types consumed weekly (based on the count of discretionary food types (takeaway, snacks, and soft drinks) consumed at least weekly) fitted with interaction terms. Adjusted ordinal models of the frequency of takeaway, snack and soft drink consumption fitted with interaction terms. All models adjusted for age, gender, children in household, relationship status, neighbourhood SES and city. IRR and OR are displayed on log scale. 20MN, 20-minute neighbourhood; IRR, incidence rate ratio; SES, socio-economic status.

The results generally showed no difference in the associations between work hours and food consumption between residents of 20MN and non-20MN (Fig. 2). However, those with a non-20MN consistently had higher odds of frequently consuming takeaway, snacks and soft drinks, and higher IRR for the number of consumed discretionary food types compared to those with a 20MN (Fig. 2). Compared to up-to-full-time workers, overtime workers had higher odds of frequently consuming takeaway if they resided in a non-20MN (OR = 1·919, 95 % CI 1·025, 3·594, *P* = 0·042) but that was not the case if they lived in a 20MN (OR = 1·060, 95 % CI 0·580, 1·937, *P* = 0·850). Non-workers had higher odds of frequently consuming snacks and soft drinks than up-to-full-time workers if they had a non-20MN (snacks OR = 1·912, 95 % CI 1·200, 3·046, *P* = 0·006, soft drinks OR = 1·660, 95 % CI 0·940, 2·931, *P* = 0·081) but not if they had a 20MN (snacks OR = 0·855, 95 % CI 0·530, 1·379, *P* = 0·521, soft drinks OR = 1·070, 95 % CI 0·582, 1·966, *P* = 0·828). Similar trends were observed when looking at combined work and commute hours among those employed (Fig. 3), except for soft drinks where the odds of frequent consumption were lower in non-20MN compared to 20MN (Fig. 3(f)). Estimates and CI from the adjusted Poisson and ordinal models are presented in Additional file 4 and 5.

## Discussion

This study examined associations between work and commute hours with food consumption and explored whether these associations were moderated by neighbourhood type. Overall, patterns suggested food behaviours tend to be less healthy for those not working and those working overtime (> 38 h/week) compared to those working up to full-time. Similarly, when considering combined work and commute hours only among those employed, results suggested those spending longer hours working and commuting were more likely to frequently eat takeaway food. When looking at the potential moderating role of neighbourhood type, the relationships between work and commute hours with food consumption appeared similar for residents of 20MN and non-20MN. However, a pattern was observed across most food behaviours, with behaviours being generally better for those in 20MN.

Findings are consistent with previous US research indicating associations between long work hours among employed parents and higher frequency of takeaway consumption^(32)^, more time spent purchasing prepared food and less time spent cooking and grocery shopping^(33)^. Limiting time and effort dedicated to food and meal preparation has been identified as a reason for purchasing takeaway meals^(34)^. Time scarcity owing to long work and commute hours may encourage takeaway consumption as a means to cope with limited time to purchase and prepare food^(5)^.

Unhealthier food behaviours among those not working compared to those working up to full-time may be linked to lower income, reducing the ability to afford healthy foods^(35)^. Although research has suggested healthy diets (as per dietary guidelines) may in fact be less expensive than current (unhealthy) diets in Australia^(36)^, low-income households still need to dedicate a bigger proportion of their household budget towards a healthy diet than high-income households^(36,37)^ and have greater concerns around food affordability than those in high-income households^(35)^. These concerns combined with the perception of healthy foods as more expensive may render it more difficult to adopt a healthy diet within a limited budget^(35)^. Alternatively, a healthy-worker effect may be at play, whereby workers are generally healthier than the general population^(38)^, having, for example, the energy to prepare and cook healthy foods^(39)^. However, additional analyses adjusting for self-rated health showed no major differences in magnitude and direction of effects (results not shown). We did not adjust for self-rated health in main analysis due to potential collider bias (where self-rated health is a common effect of exposure and outcome, distorting associations)^(40)^.

No previous research has explored the potential moderating role of 20MN in the associations between work-related time demands and food consumption. Findings of the current study suggest 20MN might have a protective role in terms of food behaviours. Higher levels of service provision and amenities may promote healthier living, aligning with projected benefits of the 20MN concept^(20)^. Increased service provision might help reduce the negative consequences of employment-related time scarcity on food consumption by providing accessible options for those with limited time to engage with their neighbourhood. However, residential neighbourhoods may only partially represent environments with which individuals frequently interact^(41)^. Time scarcity and neighbourhood features may confine individuals to defined times (e.g. opening hours) and places (e.g. types of food stores), preventing them from accessing certain places and inciting them to opt for more convenient options (e.g. drive-through takeaway outlets instead of supermarkets) or rendering it easier to access those near work during work hours or in other neighbourhoods on the way to/from work^(2)^. Food retailers near the workplace may therefore also play a role in food choice.

In addition to improving healthy food supply in residential neighbourhoods and the environments surrounding workplaces, it is also important to consider how work arrangements could be modified to deter detrimental impacts of work-related time demands on food consumption. It is possible that enabling access to flexible work hours and telecommuting, in addition to limiting long hours^(42)^, could be helpful. Previous US research has shown that those working from home spend more time preparing and consuming food at home compared to those working away from home^(43)^, with potential benefits for diet such as lower intake of calories, fat, sugar, fast-food meals and ready-to-eat meals^(44)^. However, overall, the impact of telecommuting on food practices remains largely under-investigated, warranting more research.

The important role of government in influencing what is considered full-time work has previously been recognised^(45)^. In Australia, although > 38 h per week is considered overtime^(29)^, statutory limits around additional work hours are non-existent or indicative at best^(45,46)^. Australian legislation recognises a right for workers to refuse to work ‘unreasonable’ additional hours but does not to define ‘reasonable’ additional hours, merely providing a list of factors to account for, such as the usual patterns of work in the industry and the nature of the worker’s role^(46)^. Other high-income countries such as New Zealand and the UK also lack clear regulations around overtime and maximum hours^(45)^. Regulation of limits on work hours may not directly improve food consumption but may be a step in the right direction to reduce workers’ time pressures. Additional strategies focused on healthy eating may be implemented at the organisational level such as improved availability of healthy food options at the workplace^(47)^, after all many working adults spend more than half their waking hours at work.

This is the first study to assess associations between work-related time demands and food consumption in the context of a topical urban planning policy, providing evidence on potential benefits of 20MN among those with greater work-related time demands. Categorising work hours allowed for comparisons across groups that were likely different in terms of work-related time scarcity. In addition, we examined continuous combined work and commute hours among those working, capturing a potential linear relationship with food consumption. Given commuting is generally an intrinsic part of participation in labour, combining work and commute hours enabled a more accurate assessment of work-related time demands.

Since food behaviours were self-reported, under- or over-reporting (depending on perceived social desirability) of food consumption cannot be excluded. For example, snack consumption may be underreported if snacks (e.g. chocolate, lollies, cake, chips, ice cream, donuts, sweet biscuits) are perceived as socially undesirable^(48)^. While we examined hot takeaway food such as burgers and pizza, reflecting potentially less healthy takeaway food, other takeaway food (e.g. salads) was not captured in this study. Future research should capture a wider range of takeaway options and investigate the type and healthfulness of takeaway options consumed by those with long work and commute hours. It is possible that while searching for convenience workers still frequently seek healthy takeaway alternatives. The sample size reduced power to detect smaller differences, as reflected by large CI. This also meant interpretation was based on examining patterns across work hours and neighbourhood types. While we acknowledge the response rate was under 10 %, it should have little bearing on the results. Low response rates are common for this type of recruitment approach (i.e. mass mail out to residential addresses with non-personalised invite and no individualised compensation)^(49)^. The higher number of women compared to men in the sample (61 % female) reflects the persistent gendered norm whereby women are mainly responsible for household food purchasing. The sample reflects the characteristics of main household food purchasers. No inferences are made to the wider population. While we were able to adjust for potential confounders, no information was collected on work schedules. Those with non-standard work schedules (e.g. working at night and working on weekends) may likely have different work and commute hours and poorer food behaviours^(50)^.

## Conclusion

This study suggested long work and commute hours may induce poorer food behaviours, particularly greater consumption of takeaway food. Proposed mechanisms include higher work-related time demands limiting engagement in food preparation which in turn encourages convenient options such as takeaway food. Overall, no difference in associations between work and commute hours with food consumption was found between residents of 20MN and non-20MN. However, patterns suggested 20MN may, through improved service and amenities provision, benefit some aspects of workers’ food consumption and potentially attenuate negative impacts of their work-related time demands. Opportunities exist to further explore the potential moderating role of 20MN in links between work-related time demands and food practices, examining the location of workers’ food practices, for example, whether food purchasing occurs close to home or close to work.

## Supplementary material

For supplementary material accompanying this paper visit https://doi.org/10.1017/S1368980023000587. click here to view supplementary material
