# Do 20-minute neighbourhoods moderate associations between work and commute hours with food consumption? – ADDENDUM

**DOI:** 10.1017/S1368980024000314

**Published:** 2024-02-08

**Authors:** Laura Helena Oostenbach, Karen Elaine Lamb, David Crawford, Anna Timperio, Lukar Ezra Thornton

Some funding information was not included in the above published article. The acknowledgements section has now been updated to include the following:

“This work was supported by an Australian Research Council Discovery Project Grant [DP 170100751] to LET and KEL. The Australian Research Council had no role in the design, analysis or writing of this article.”
