# Supplementary material for: Do 20-minute neighbourhoods moderate associations between work and commute hours with food consumption?
Source: Public Health Nutr. 2023 Mar 29;26(10):2026–35. doi: 10.1017/S1368980023000587 (PMC10564605; doi:10.1017/S1368980023000587)
Supplement: Supplementary file 1 [file S1368980023000587sup001.pdf]

Additional file 1: Variables included in the study and their respective ProjectPLAN question

Variable	Question in ProjectPLAN	Categories in ProjectPLAN	Derivation process	Final response
**Fruit**	*How many serves of fruit do you usually eat each day? Include fresh, dried, frozen and tinned fruit.*<br><br>*Below are examples of ONE serve of fruit:*<br>*1 medium sized fruit e.g. apple, banana or pear*<br>*2 small sized fruits e.g. apricots, kiwi fruits or plums*<br>*1 cup fruit e.g. diced or canned*	(0) I don't eat fruit<br>(0.5) Less than one serve per day<br>(1) 1 serve per day<br>(2) 2 serves per day<br>(3) 3 serves per day<br>(4) 4 serves per day<br>(5) 5 serves per day<br>(6) 6 serves per day<br>(7) 7 serves per day<br>(8) 8 or more serves per day	"Less than one serve per day" was considered half a serve of fruit per day and treated as a numeric value of 0.5. "8 or more" was treated as a numeric value of 8.	0-8 serves per day
**Vegetables**	*How many serves of vegetables do you usually eat each day? Include fresh, frozen and tinned vegetables.*<br><br>*Below are examples of ONE serve of vegetables:*	(0) I don't eat vegetables<br>(0.5) Less than one serve per day<br>(1) 1 serve per day<br>(2) 2 serves per day<br>(3) 3 serves per day<br>(4) 4 serves per day<br>(5) 5 serves per day	"Less than one serve per day" was considered half a serve of vegetables per day and treated as a numeric value of 0.5. "10 or more" was treated as a numeric value of 10.	0-10 serves per day

	½ cup vegetables e.g. broccoli, spinach, carrots or pumpkin<br>*½ cup beans or lentils*<br>*1 cup green leafy or raw salad vegetables*	(6) 6 serves per day<br>(7) 7 serves per day<br>(8) 8 serves per day<br>(9) 9 serves per day<br>(10) 10 or more serves per day		
**Takeaway**	*How often would you usually consume hot take-away food like fish and chips, burgers, pizza, sausage rolls, meat pies, fried chicken?*	(0) Never or less than once per month<br>(0.5) 1 - 3 times per month<br>(1) Once per week<br>(2) Twice per week<br>(3) 3 times per week<br>(4) 4 times per week<br>(5) 5 times per week<br>(6) 6 times per week<br>(7) 7 or more times per week	Categories 0 and 0.5 were combined into one category. Categories 2 to 7 were combined into one category.	(0) Less than weekly<br>(1) Once per week<br>(2) More than once per week
**Snack**	*How many days per week would you usually consume snacks like chocolate, lollies, cake, a packet of chips, ice cream, donuts, sweet biscuits?*	(0) Never or less than once per month<br>(0.5) 1 - 3 times per month<br>(1) Once per week<br>(2) Twice per week<br>(3) 3 times per week<br>(4) 4 times per week	Categories 0 and 0.5 were combined into one category. Categories 1 and 2 were combined into one category. Categories 3 and 4 were combined into one category.	(0) Less than weekly<br>(1) Once or twice per week<br>(2) 3 or 4 times per week<br>(3) 5 or more times per week

		(5) 5 times per week<br>(6) 6 times per week<br>(7) 7 or more times per week	Categories 5 to 7 were combined into one category.	
**Soft drink**	*How many days per week would you usually consume sugar-sweetened beverages like soft drinks, energy drinks, fruit juice, iced tea, sports drinks or cordial? This does not include diet varieties*	(0) Never or less than once per month<br>(0.5) 1 - 3 times per month<br>(1) Once per week<br>(2) Twice per week<br>(3) 3 times per week<br>(4) 4 times per week<br>(5) 5 times per week<br>(6) 6 times per week<br>(7) 7 or more times per week	Categories 0 and 0.5 were combined into one category. Categories 1 and 2 were combined into one category. Categories 3 to 7 were combined into one category.	(0) Less than weekly<br>(1) Once or twice per week<br>(2) 3 or more times per week
**Number of different types of discretionary food consumed weekly**	N/A	N/A	1. A binary variable was first computed for each type of discretionary food item (takeaway, snack, soft drink): (0) consumed less than weekly and (1) consumed weekly.<br><br>2. A total count variable was then derived, representing the	0-3 discretionary food items (amongst takeaway, snack, and soft drink) consumed at least weekly

<table>
<tr><td></td><td></td><td></td><td>number of items (from takeaway, snack, and soft drink) consumed at least weekly.</td><td></td></tr>
<tr><td rowspan="2">Work hours (categories)</td><td>In a usual week, which of the following describes your current activities and/or responsibilities?
Employed in a paid job (including self-employed)</td><td>(0) No
(1) Yes</td><td rowspan="2">Those who reported not being employed were categorised into one category.

Those working up to full-time, i.e., ≤38 hours/week as per Fair Work's definition, were combined into one category.

Those working overtime, i.e. >38 hours/week as per Fair Work's definition, were combined into one category.</td><td rowspan="2">(0) Not working
(1) Working up to full-time
(2) Working overtime</td></tr>
<tr><td>Respondents who reported being employed were asked about their work hours.

In a normal week, how many hours per week do you work in all your paid jobs?</td><td>Hours per week</td></tr>
</table>

**Work hours**	Respondents who reported being employed were asked about their work hours.<br><br>*In a normal week, how many hours per week do you work in all your paid jobs?*	Hours per week	N/A	Hours per week
**Commute hours**	*Thinking about your main paid job, which of the following best describes the location of your workplace?*	(1) I usually work from home<br>(2) I usually travel to the same work location on the days I work<br>(3) I usually travel to many different work locations on the days I work e.g., tradesperson or courier driver	1. Daily commute minutes for each commute way (from home to work and vice versa) were derived based on the hours and minutes reported. “3 or more hours” was treated as a numeric value of 3. Hours were multiplied by 60 to represent minutes. Those (transformed) minutes were then added to the reported minutes.<br><br>2. Daily commute minutes for both commute ways were	Hours per week
	Respondent who reported usually travelling to the same work location or to many different work locations were asked about their commute time.	<u>Hours:</u><br>(0) 0 hours<br>(1) 1 hour<br>(2) 2 hours<br>(3) 3 or more hours		

	On a usual day, how long is your travel time: in hours and/or minutes<br>a. *From home to work*<br>b. *From work to home*	<u>Minutes:</u><br>(0) 0 mins<br>(5) +5 mins<br>(10) +10 mins<br>(15) +15 mins<br>…<br>(55) +55 mins	summed into one commute time (minutes).<br><br>3. To represent weekly commute minutes, commute minutes were multiple by the number of reported workdays. "Less than once per week" was treated as a numeric value of 1.<br><br>4. To represent weekly commute hours, weekly commute minutes were divided by 60.<br><br>5. Those who reported usually working from home were transformed into 0 weekly commute hours instead of missing.	
	In a normal week, how many days per week do you work in all your paid jobs?	(0.5) Less than once per week<br>(1) One day<br>(2) Two days<br>(3) Three days<br>(4) Four days<br>(5) Five days<br>(6) Six days<br>(7) Seven days per week (every day)		
**Combined work and commute hours**	N/A	N/A	Both work and commute hours were summed.	Hours per week

**Age**	*What is your current age in years?*	Age in years	N/A	Years
**Gender**	*What is your gender?*	(1) Male<br>(2) Female<br>(3) Transgender	N/A	(1) Male<br>(2) Female<br>(3) Transgender
**Presence of children in household**	*<u>Not including you</u>, how many other people live in your household <u>most nights of the week</u>?*<br>*a. Children (4 years or younger)*<br>*b. Children (5 to 12 years)*<br>*c. Children (13 to 17 years)*	(0) 0 people<br>(1) 1 person<br>(2) 2 people<br>(3) 3 people<br>(4) 4 people<br>(5) 5 people<br>(6) 6 or more people	Categories were created based on the presence of any children in the household and whether any 4 years old or younger or whether all children were older than 4 years old.	(0) No children<br>(1) Any child ≤ 4 years<br>(2) Only children aged 5 to 17 years
**Relationship/living status**	*What is your current relationship status?*	(1) Single<br>(2) In a relationship living with partner<br>(3) In a relationship not living with partner	Categories 2 and 3 were recoded.	(1) Single<br>(2) In a relationship not living with partner<br>(3) In a relationship living with partner
**Neighbourhood SES**	N/A<br>Stratified sampling based on neighbourhood SES.	(0) Low SES<br>(1) High SES	N/A	(0) Low SES<br>(1) High SES

	Low based on Statistical Areas level 1 (SA1) Socio-Economic Indexes for Areas (SEIFA) Index of Relative Socio-economic Advantage and Disadvantage (IRSAD) deciles of level 1, 2 or 3 that had to be within an SA2 of level 1, 2 or 3. (27)<br><br>High based on SA1 SEIFA IRSAD deciles of level 8, 9 or 10 that had to be within an SA2 of level 8, 9 or 10. (27)			
**Neighbourhood type**	N/A<br>Stratified sampling based on neighbourhood type.<br><br>Five layers of resources were identified, including: 1) healthy food; 2) community facilities; 3) recreation facilities; 4)	(0) 20-minute neighbourhood<br>(1) Non-20-minute neighbourhood	N/A	(1) 20-minute neighbourhood<br>(2) Non-20-minute neighbourhood

	public open space; and 5) public transport. Twenty-minute neighbourhoods were areas with access to all five layers of resources, whilst non-20MNs had very few individual services and amenities (≤ 5 individual attributes). (Thornton, under review).			
**City**	N/A<br>Stratified sampling based on city.	(1) Melbourne<br>(2) Adelaide	N/A	(1) Melbourne<br>(2) Adelaide

N/A: not applicable

Additional file 2: Descriptive characteristics for the full sample, complete case sample and omitted participants

	Whole sample			Employed sub-sample		
	**Omitted**	**Complete**	**Full**	**Omitted**	**Complete**	**Full**
**N**	70	699	769	28	378	406
**Fruit consumption (serves/day)** median (IQR)	2.00 (1.00, 2.00) (n=70)	1.00 (1.00, 2.00) (n=699)	1.00 (1.00, 2.00) (n=769)	2.00 (1.00, 2.00) (n=28)	2.00 (1.00, 2.00) (n=378)	2.00 (1.00, 2.00) (n=406)
**Vegetable consumption (serves/day)** median (IQR)	2.00 (1.00, 3.00) (n=70)	2.00 (1.00, 3.00) (n=699)	2.00 (1.00, 3.00) (n=769)	2.00 (1.00, 3.00) (n=28)	2.00 (2.00, 3.00) (n=378)	2.00 (2.00, 3.00) (n=406)
**Different types of discretionary food (i.e., takeaway, snacks, and soft drinks) consumed weekly (n)**						
0	16 (23.2%)	137 (19.6%)	153 (19.9%)	4 (14.3%)	67 (17.7%)	71 (17.5%)
1	35 (50.7%)	285 (40.8%)	320 (41.7%)	13 (46.4%)	144 (38.1%)	157 (38.7%)
2	11 (15.9%)	166 (23.7%)	177 (23.0%)	6 (21.4%)	94 (24.9%)	100 (24.6%)
3	7 (10.1%)	111 (15.9%)	118 (15.4%)	5 (17.9%)	73 (19.3%)	78 (19.2%)
**Takeaway consumption (occasions)**						
<1/week	54 (78.3%)	470 (67.2%)	524 (68.2%)	19 (67.9%)	224 (59.3%)	243 (59.9%)
1/week	9 (13.0%)	139 (19.9%)	148 (19.3%)	6 (21.4%)	91 (24.1%)	97 (23.9%)
>1/week	6 (8.7%)	90 (12.9%)	96 (12.5%)	3 (10.7%)	63 (16.7%)	66 (16.3%)
**Snack consumption (occasions)**						
<1/week	17 (24.3%)	189 (27.0%)	206 (26.8%)	4 (14.3%)	97 (25.7%)	101 (24.9%)

1-2/week	24 (34.3%)	204 (29.2%)	228 (29.6%)	11 (39.3%)	119 (31.5%)	130 (32.0%)
3-4/week	17 (24.3%)	131 (18.7%)	148 (19.2%)	6 (21.4%)	73 (19.3%)	79 (19.5%)
≥5/week	12 (17.1%)	175 (25.0%)	187 (24.3%)	7 (25.0%)	89 (23.5%)	96 (23.6%)
**Soft drink consumption (occasions)**						
<1/week	58 (82.9%)	488 (69.8%)	546 (71.0%)	21 (75.0%)	262 (69.3%)	283 (69.7%)
1-2/week	5 (7.1%)	107 (15.3%)	112 (14.6%)	3 (10.7%)	62 (16.4%)	65 (16.0%)
≥3/week	7 (10.0%)	104 (14.9%)	111 (14.4%)	4 (14.3%)	54 (14.3%)	58 (14.3%)
**Work hours**						
Not working (0h)	9 (28.1%)	321 (45.9%)	330 (45.1%)		0 (0.0%)	
Working up to full-time (1-38h)	14 (43.8%)	237 (33.9%)	251 (34.3%)	14 (60.9%)	237 (62.7%)	251 (62.6%)
Working overtime (>38h)	9 (28.1%)	141 (20.2%)	150 (20.5%)	9 (39.1%)	141 (37.3%)	150 (37.4%)
**Combined weekly work and commute hours** (employed only) median (IQR)	44.42 (27.83, 50.00) (n=22)	40.46 (27.50, 47.50) (n=378)	40.67 (27.62, 47.50) (n=400)	44.42 (27.83, 50.00) (n=22)	40.46 (27.50, 47.50) (n=378)	40.67 (27.62, 47.50) (n=400)
**Weekly work hours** (employed only) median (IQR)	38.00 (20.00, 40.00) (n=23)	37.50 (25.00, 40.00) (n=378)	37.50 (25.00, 40.00) (n=401)	38.00 (20.00, 40.00) (n=23)	37.50 (25.00, 40.00) (n=378)	37.50 (25.00, 40.00) (n=401)
**Weekly commute hours** (employed only) median (IQR)	4.00 (0.00, 7.50) (n=26)	3.33 (1.50, 6.25) (n=378)	3.33 (1.33, 6.25) (n=404)	4.00 (0.00, 7.50) (n=26)	3.33 (1.50, 6.25) (n=378)	3.33 (1.33, 6.25) (n=404)
**Age** median (IQR)	68.00 (53.00, 73.50) (n=32)	57.00 (41.00, 67.00) (n=699)	58.00 (41.00, 67.00) (n=731)	53.00 (45.00, 68.00) (n=7)	47.00 (36.00, 57.00) (n=378)	47.00 (37.00, 57.00) (n=385)
**Gender**						

Male	26 (43.3%)	270 (38.6%)	296 (39.0%)	12 (44.4%)	140 (37.0%)	152 (37.5%)
Female	34 (56.7%)	427 (61.1%)	461 (60.7%)	15 (55.6%)	238 (63.0%)	253 (62.5%)
Transgender	0 (0.0%)	2 (0.3%)	2 (0.3%)		0 (0.0%)	
**Children in household**						
No children	43 (69.4%)	514 (73.5%)	557 (73.2%)	17 (60.7%)	248 (65.6%)	265 (65.3%)
Any child ≤ 4 years	14 (22.6%)	97 (13.9%)	111 (14.6%)	8 (28.6%)	61 (16.1%)	69 (17.0%)
Only children aged 5 to 17 years	5 (8.1%)	88 (12.6%)	93 (12.2%)	3 (10.7%)	69 (18.3%)	72 (17.7%)
**Relationship status**						
Single	22 (39.3%)	210 (30.0%)	232 (30.7%)	11 (45.8%)	114 (30.2%)	125 (31.1%)
In a relationship: not living with partner	1 (1.8%)	43 (6.2%)	44 (5.8%)	0 (0.0%)	24 (6.3%)	24 (6.0%)
In a relationship: living with partner	33 (58.9%)	446 (63.8%)	479 (63.4%)	13 (54.2%)	240 (63.5%)	253 (62.9%)
**Neighbourhood SES**						
Low SES	30 (42.9%)	307 (43.9%)	337 (43.8%)	15 (53.6%)	159 (42.1%)	174 (42.9%)
High SES	40 (57.1%)	392 (56.1%)	432 (56.2%)	13 (46.4%)	219 (57.9%)	232 (57.1%)
**City**						
Melbourne	38 (54.3%)	320 (45.8%)	358 (46.6%)	17 (60.7%)	189 (50.0%)	206 (50.7%)
Adelaide	32 (45.7%)	379 (54.2%)	411 (53.4%)	11 (39.3%)	189 (50.0%)	200 (49.3%)
**Neighbourhood design**						
20MN	29 (41.4%)	349 (49.9%)	378 (49.2%)	9 (32.1%)	199 (52.6%)	208 (51.2%)
Non-20MN	41 (58.6%)	350 (50.1%)	391 (50.8%)	19 (67.9%)	179 (47.4%)	198 (48.8%)

Additional file 3: Adjusted models of work hours and food consumption (n=699)

	**Reference group:** working up to full-time			
Poisson regression		**IRR**	**95% CI**	**p-value**
Daily serves of fruit	Not working	0.920	(0.790; 1.072)	0.286
	Working overtime	0.929	(0.782; 1.103)	0.402
Daily serves of vegetables	Not working	0.912	(0.806; 1.032)	0.145
	Working overtime	1.065	(0.932; 1.217)	0.353
Number of different types of discretionary food (i.e., takeaway, snacks, and soft drinks) consumed weekly	Not working	1.071	(0.906; 1.265)	0.424
	Working overtime	1.103	(0.926; 1.313)	0.274
Ordinal regression		**OR**	**95% CI**	**p-value**
Takeaway consumption	Not working	1.131	(0.732; 1.750)	0.579
	Working overtime	1.410	(0.908; 2.190)	0.126
Snack consumption	Not working	1.308	(0.915; 1.868)	0.140
	Working overtime	1.156	(0.791; 1.688)	0.454
Soft drink consumption	Not working	1.352	(0.872; 2.096)	0.177
	Working overtime	1.303	(0.819; 2.073)	0.263

Models adjusted for age, gender, children in household, relationship status, neighbourhood SES, neighbourhood type and city.

(Not working: 0h, Up to full-time: 1-38h/week, Overtime: >38h/week)

IRR=Incidence Rate Ratio, OR=Odds Ratio, CI=Confidence Interval.

Additional file 4: Adjusted models of work hours and food consumption (n=699)

	**Reference group:** working up to full-time	**20MN**			**Non-20MN**		
Poisson regression		**IRR**	**95% CI**	**p-value**	**IRR**	**95% CI**	**p-value**
Daily serves of fruit	Not working	0.870	(0.710; 1.065)	0.176	0.969	(0.793; 1.185)	0.761
	Working overtime	0.938	(0.747; 1.177)	0.578	0.915	(0.706; 1.185)	0.500
Daily serves of vegetables	Not working	0.866	(0.733; 1.023)	0.090	0.954	(0.812; 1.120)	0.564
	Working overtime	1.086	(0.911; 1.295)	0.356	1.034	(0.846; 1.263)	0.746
Number of different types of discretionary food (i.e., takeaway, snacks, and soft drinks) consumed weekly	Not working	0.944	(0.748; 1.193)	0.631	1.192	(0.960; 1.481)	0.112
	Working overtime	1.016	(0.799; 1.291)	0.898	1.203	(0.937; 1.544)	0.146
Ordinal regression		**OR**	**95% CI**	**p-value**	**OR**	**95% CI**	**p-value**
Takeaway consumption	Not working	0.893	(0.483; 1.653)	0.719	1.401	(0.798; 2.460)	0.241
	Working overtime	1.060	(0.580; 1.937)	0.850	1.919	(1.025; 3.594)	0.042
Snack consumption	Not working	0.855	(0.530; 1.379)	0.521	1.912	(1.200; 3.046)	0.006
	Working overtime	1.106	(0.660; 1.854)	0.702	1.204	(0.696; 2.082)	0.506
Soft drink consumption	Not working	1.070	(0.582; 1.966)	0.828	1.660	(0.940; 2.931)	0.081
	Working overtime	1.088	(0.578; 2.047)	0.795	1.584	(0.813; 3.087)	0.176

Models adjusted for age, gender, children in household, relationship status, neighbourhood SES and city.

(Not working: 0h, Up to full-time: 1-38h/week, Overtime: >38h/week)

20MN=20-minute neighbourhood, IRR=Incidence Rate Ratio, OR=Odds Ratio, CI=Confidence Interval

Additional file 5: Adjusted models of combined work and commute hours and food consumption amongst those employed (n=378)

	**Combined work and commute hours**					
	**20MN**			**Non-20MN**		
Poisson regression	**IRR**	**95% CI**	**p-value**	**IRR**	**95% CI**	**p-value**
Daily serves of fruit	0.998	(0.991; 1.006)	0.645	0.996	(0.988; 1.004)	0.304
Daily serves of vegetables	1.002	(0.996; 1.008)	0.445	1.000	(0.994; 1.006)	0.992
Number of different types of discretionary food (i.e., takeaway, snacks, and soft drinks) consumed weekly	1.003	(0.995; 1.011)	0.512	1.006	(0.998; 1.014)	0.148
Ordinal regression	**OR**	**95% CI**	**p-value**	**OR**	**95% CI**	**p-value**
Takeaway consumption	1.011	(0.990; 1.033)	0.315	1.018	(0.996; 1.040)	0.106
Snack consumption	1.004	(0.986; 1.023)	0.640	1.014	(0.996; 1.032)	0.132
Soft drink consumption	1.015	(0.992; 1.038)	0.212	1.005	(0.983; 1.028)	0.648

Models adjusted for age, gender, children in household, relationship status, neighbourhood SES and city.

(Not working: 0h, Up to full-time: 1-38h/week, Overtime: >38h/week)

20MN=20-minute neighbourhood, IRR=Incidence Rate Ratio, OR=Odds Ratio, CI=Confidence Interval.
